# The Effects of Intravenous Lipid Emulsion Therapy in the Prevention of Depressive Effects of Propofol on Cardiovascular and Respiratory Systems: An Experimental Animal Study

**DOI:** 10.3390/medicina55010001

**Published:** 2018-12-25

**Authors:** Fatih Doğanay, Rohat Ak, Halil Alışkan, Serdar Abut, Engin Sümer, Özge Onur

**Affiliations:** 1 Department of Emergency Medicine, Erciş State Hospital, Van 65400, Turkey; 2 Department of Emergency Medicine, Şehit Prof. Dr. İlhan Varank Sancaktepe Education and Research Hospital, Istanbul 34885, Turkey; rohatakmd@gmail.com; 3 Department of Emergency Medicine, Şişli Hamidiye Etfal Education and Research Hospital, Istanbul 34381, Turkey; halilaliskan@gmail.com; 4 The Institute of Natural and Applied Sciences, Van Yüzüncü Yıl University, Van 65100, Turkey; serdarabut@yyu.edu.tr; 5 Department of Pharmaceutical Toxicology, Graduate School of Health Sciences, Yeditepe University, Istanbul 34752, Turkey; vetenginsumer@gmail.com; 6 Department of Emergency Medicine, Marmara University Faculty of Medicine, Istanbul 34916, Turkey; ozberkozge@gmail.com

**Keywords:** acute toxicity, cardiovascular depression, intravenous lipid emulsion, propofol, rat model, respiratory depression

## Abstract

*Background and objectives*: Although there are several hypotheses about the mechanism of action, intravenous lipid emulsion (ILE) therapy has been shown to be effective in the treatment of toxicities due to local anaesthetics and many lipophilic drugs. In this study, we had hypothesized that ILE therapy might also be effective in preventing mortality and cardiorespiratory depressant effects due to propofol intoxication. *Materials and methods*: Twenty-eight Sprague-Dawley adult rats were randomly divided into four groups. Saline was administered to the subjects in the control group. The second group was administered propofol (PP group); the third group was administered ILE (ILE group), and the fourth group was administered propofol and ILE therapy together (ILE+PP group). Systolic blood pressure (SBP), diastolic blood pressure (DBP), respiratory rate (RR), heart rate (HR), and mortality were recorded at 10 time-points during a period of 60 min. A repeated measures linear mixed-effect model with unstructured covariance was used to compare the groups. *Results*: In the PP group; SBP, DBP, RR, and HR levels declined steadily; and all rats in this group died after the 60-min period. In the ILE+PP group, the initially reduced SBP, DBP, RR, and HR scores increased close to the levels observed in the control group. The SBP, DBP, RR, and HR values in the PP group were significantly lower compared to the other groups (*p* < 0.01). The mortality rate was 100% (with survival duration of 60 min) for the PP group; however, it was 0% for the remaining three groups. *Conclusions*: Our results suggest that the untoward effects of propofol including hypotension, bradycardia, and respiratory depression might be prevented with ILE therapy.

## 1. Introduction

Propofol is a general anesthetic used for induction and maintenance of anesthesia. It is also commonly used for sedation purposes [1]. Propofol is generally safe but it may cause severe side effects such as cardiovascular depression and hypotension. Propofol causes hypotension most commonly by lowering systemic vascular resistance, cardiac contractility and cardiac preload. There is no known antidote for propofol today.

There are several studies examining the cardiac depressant effects of propofol in the literature. In one of those studies, the incidence of hypotension (SBP < 90 mmHg) and bradycardia (HR < 50 pulse/min) were developed in 15.7% and 4.8% of the patients respectively after receiving an induction dose propofol. The same study reported that 77% of the hypotension attacks and 42% of these bradycardia attacks occurred within the first 10 min [2]. Abdul Zahoor et al. reported reductions in the blood pressure and heart rate after propofol application in patients undergoing minor surgeries [3]. The study by Ebert et al. on healthy volunteers compared the effects of propofol and placebo; and significant reductions in the respiratory rate, blood pressure, and pulse rate were reported in the propofol group [4].

Intravenous lipid emulsion (ILE) therapy was first introduced with the aim of preventing the toxic effects of local anesthetics [5]. Weinberg et al. reported that ILE was an effective therapy for hemodynamic recovery in dogs and resuscitated rats after an overdose of bupivacaine [5,6]. In later studies, it was shown that ILE could be used as an effective antidote in poisoning with lipophilic drugs [7]. ILE therapy has been used successfully in toxicities due to many lipophilic drugs including beta-blockers [8], calcium channel blockers [9], and tricyclic antidepressants [10], as well as, the local anesthetics [11]. A case report by Tsui et al. stated that vasopressor-resistant hypotension and hypotension resistant to fluid therapy were recovered with ILE therapy after post-epidural anesthesia [12]. It is also suggested that ILE can be effective in reversing the respiratory depression, vasodilatation, and cardiac depressant effects of propofol owing to its high lipophilicity.

The primary aim of this study was to assess the effects of ILE therapy on propofol toxicity in terms of survival. The secondary aim was to assess the effects of ILE therapy in preventing the depressive effects of propofol on the cardiovascular and respiratory systems.

## 2. Materials and Methods

The study was approved by the Ethical Committee of Yeditepe University, Medical Faculty, Experimental Animals Research Laboratory (Atasehir, Istanbul, Turkey34755) on 5 April 2016 with the registry number 529. Authors declare here that all applicable international, national and/or institutional guidelines for the care and use of animals were followed. All procedures and practices involving animals in the study were in compliance with the ethical standards of the respective institution at which the study was conducted.

The consent for this study was taken from the ethical board of experimental animals, and the ethics committee allowed an upper limit of 28 rats to be included in the study.

### 2.1. Subject Selection

A total of 28 Sprague-Dawley adult rats weighing in the range from 200 to 300 g were included in the study. All experimental animals were kept in a day and night cycle for 12 h each and at 24 ± 4 °C for seven days until the start of the experiment, providing their adaptation to the environment. Standard diet and water were given to the rats.

### 2.2. Experiment Protocol and Groups

Hair in the lower parts of the bodies of the rats was shaved to perform the applications easily. The rats were kept in supine position with their extremities stabilized during the administration of the study processes.

Isoflurane inhalation anesthesia (Isoflurane, Isofludem 100 mL) was used in all rats with an animal anesthesia machine. After ensuring a satisfactory depth of anesthesia, femoral artery cannulations were performed in all groups using a 26-G catheter (i.v. NEO ALPHA, La-med Healthcare, Haryana, India). Using a disposable pressure transducer set (OKUMAN Medikal Sistemler Ltd. Şti., Ankara, Turkey) and a standard cardiac monitor (NIHON KOHDEN Cardiolife Monitor TEC-7721 K, Nihon Kohden Corporation, Tokyo, Japan), a momentary blood pressure monitoring was performed. Four rats died due to the complications during the application of femoral artery cannulation. The study was conducted with six rats in each group.

Electrocardiography electrodes were placed on both the forefeet and right hind legs of the study rats. Respiratory and pulse rates were monitored with the same device (NIHON KOHDEN Cardiolife Monitor TEC-7721 K, Nihon Kohden Corporation, Tokyo, Japan).

Tail vein cannulation was made with a 26-G cannula (i.v. NEO ALPHA, La-med Healthcare, Hayrana, India) in all groups to administer the study medications. Propofol (PROPOFOL-LIPURO1%, 10 mg/mL, I.V. 20 Ml Ampul for Infusion, İlaçsan^®^/Turkey), ILE (ClinOleic 20% lipid 500 mL, ECZACIBAŞI Baxter/Belgium), and 0.9% NaCl were administered with an infusion pump (Swiss Made, Arcomed AG Volumed VP 7000, Kloten, Switzerland). The medication dosages and administration methods were determined and the experimental protocol was designed based on the study reports in the literature and references explained previously [8,13,14].

Rats were randomly assigned to four equal groups as follows:

Group 1 (n = 6) was the control group, in which a 0.9% NaCl was administered as a four-minute infusion in four minutes with a dose of 16 mL/kg.

Group 2 (n = 6) was the PP group, in which propofol was applied as an i.v. bolus with a dose of 42 mg/kg in the fifth minute after the pre-treatment with 0.9% NaCI as a pro-drug as described in Group 1.

Group 3 (n = 6) was the ILE group, in which a four-minute ILE infusion was administered at a dose of 16 mL/kg.

Group 4 (n = 6) was the ILE+PP group, in which ILE was applied as a pro-drug as described in Group 3. After this pre-treatment with ILE, propofol was applied in the fifth minute as an i.v. bolus at a dose of 42 mg/kg.

At 10 different time-points in the first 60 min (minutes 0, 1, 3, 5, 6, 8, 10, 20, 30, and 60), the following parameters were measured in each rat, including the systolic blood pressure (SBP), diastolic blood pressure (DBP), respiratory rate (RR), and the heart rate (HR). Each rat was monitored during this period of 60 min to observe whether any cardiac or respiratory arrests would occur. After the administration of study medications, the time-points when mortality took place were recorded. All rats were decapitated with guillotine under anesthesia in the 60th min.

### 2.3. Statistical Examinations

Trend analysis approach examines the relationship between the treatment order and response size. Trend analyses in clinical studies focus on causal treatment effects rather than simple comparisons [15]. A total of 240 data points were available for statistical analyses in this present study (24 rats × 10 different time points). Of the 24 rats, only 18 had complete data because of the missing values due to the death of the rats in the PP group. All rats in the PP group died at different time-points in the first 60 min. This resulted in 15 out of 240 (6%) time points to evaluate instead of the ratio of 6 rats out of 24 (25%). The main aim of the statistical analyses was to demonstrate the significant differences between the repeated measures in the four experimental groups. Due to the unbalanced data structure, we aimed to use the linear mixed-effect model (LMM) to compare the groups [16]. The different values in the groups were compared using an unstructured covariance linear mixed model. *p* values <0.05 were considered to be statistically significant. All mixed model statistical analyses were conducted using a SAS 9.4 (SAS Institute, Cary, NC, USA).

## 3. Results

**Figure 1:**
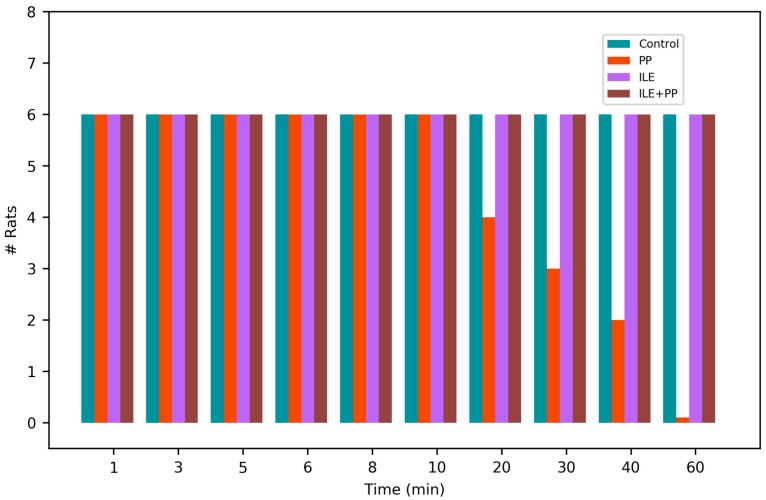
Number of living or dead rats in the groups over time.

In the PP group, cardiac arrests occurred in each rat at different time points; in the 15th, 20th, 25th, 40th, 44th, and 55th min respectively (Figure 1). No mortalities were observed in the other study groups.

**Figure 2:**
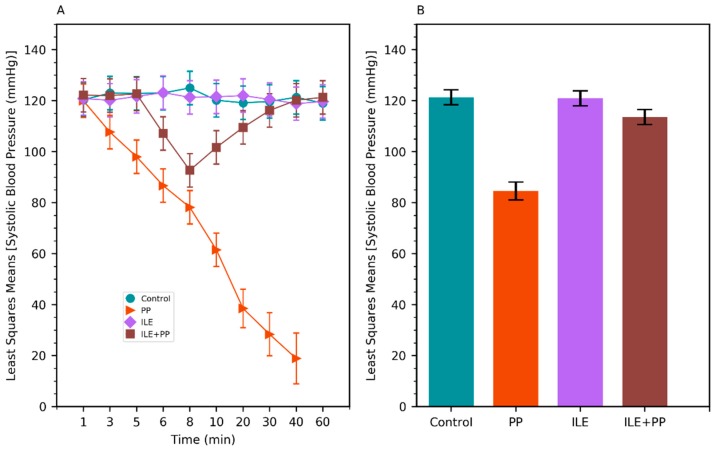
(**A**) Least squares means of systolic blood pressure (SBP) values of Control, PP, ILE, and ILE+PP groups of rats versus time. (**B**) Least squares means of SBP values (Error bars: 95% confidence interval).

**Figure 3:**
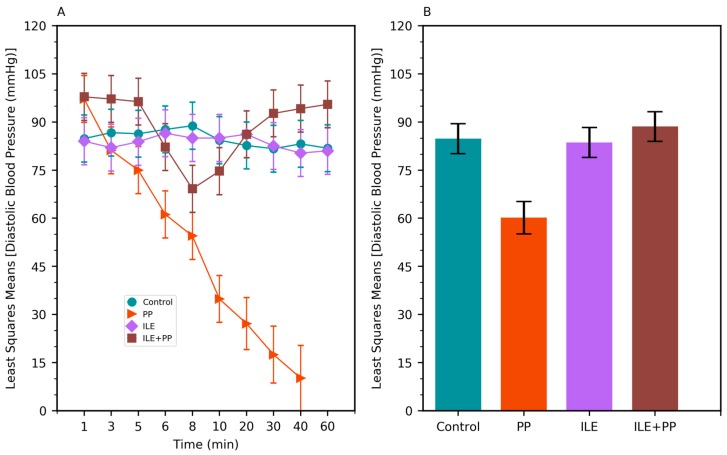
(**A**) Least squares means of diastolic blood pressure (DBP) values of Control, PP, ILE, and ILE+PP groups of rats versus time. (**B**) Least squares means of DBP values (Error bars: 95% confidence interval).

**Figure 4:**
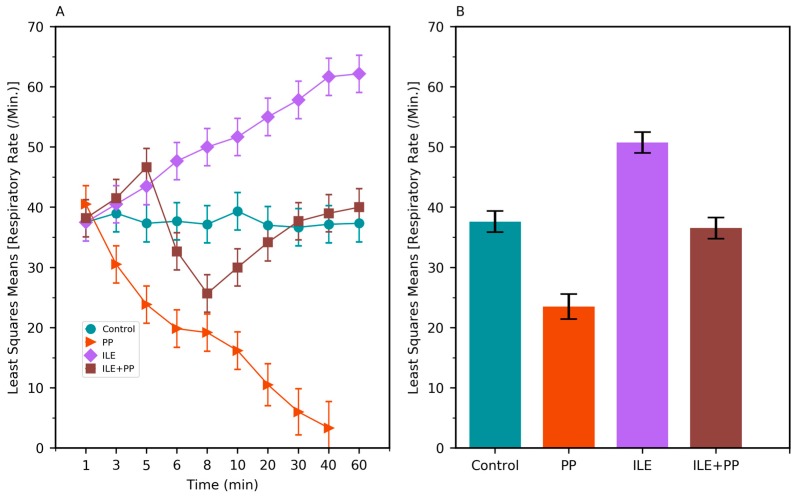
(**A**) Least squares means of the respiratory rate (RR) values of Control, PP, ILE, and ILE+PP groups of rats versus time. (**B**) Least squares means of RR values (Error bars: 95% confidence interval).

**Figure 5:**
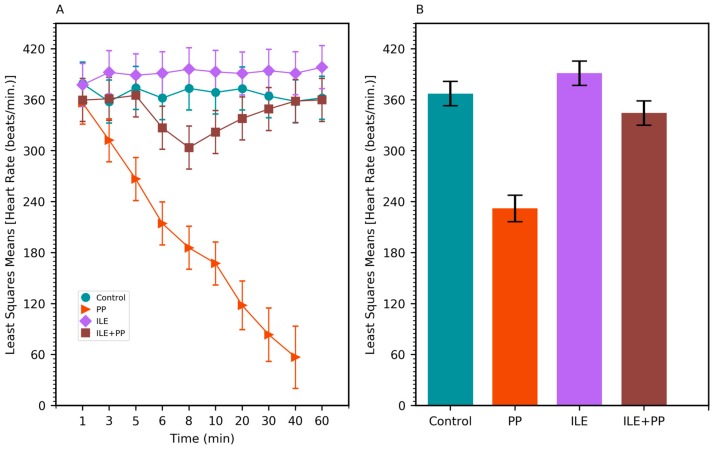
(**A**) Least squares means of the heart rate (HR) values of Control, PP, ILE, and ILE+PP groups of rats versus time. (**B**) Least squares means of HR values (Error bars: 95% confidence interval).

A linear mixed model was conducted to compare the systolic blood pressure (SBP), diastolic blood pressure (DBP), respiratory rate (RR), and heart rate (HR) values between groups. SBP, DBP, RR, and HR values in the PP group were found to be lower than those of the Control, ILE and ILE+PP groups and *p* values were <0.0001 (*p* < 0.05) (Table 1, Figure 2B, Figure 3B, Figure 4B and Figure 5B).

The pairwise comparisons demonstrated that RR values of the ILE group were higher than those of the Control group (mean difference = 13.13/min, *p* < 0.0001). The differences between SBP, DBP, and HR values in the Control and ILE groups were not significant and p values were 1.00, 1.00, and 0.183 respectively (*p* > 0.05) (Table 1, Figure 2B, Figure 3B, Figure 4B and Figure 5B).

SBP values of the ILE+PP group were found to be significantly lower than the Control group (mean difference = 7.80 mmHg, *p* = 0.009). The differences between DBP, RR, and HR values in the ILE+PP and Control groups were not significant and p values were 1.00, 1.00, and 0.234 respectively (*p* > 0.05) (Table 1, Figure 2B, Figure 3B, Figure 4B and Figure 5B).

SBP, RR, and HR values of the ILE group were higher than those of the ILE+PP group. Mean differences were 7.42, 14.20, and 46.93 with *p* values of *p* = 0.013, *p* < 0.0001, and *p* = 0.0012, respectively. The differences in DBP in the ILE and ILE+PP groups were not significant (*p* = 0.937, *p* > 0.05) (Table 1, Figure 2B, Figure 3B, Figure 4B and Figure 5B).

## 4. Discussion

Cardiac side effects of propofol are related to the blockage of the voltage-gated sodium (Na) and potassium (K) channels at the molecular level [17,18]. Propofol stimulates the release of nitric oxide, blocks the calcium channels, and activates protein kinase C [19,20]. Therefore, administration of propofol results in a reduction in the cardiac output and arterial blood pressure. [2,21]. Propofol suppresses the baroreceptor reflex and decreases sympathetic nerve activity. In previous studies, it was reported that propofol caused the emergence of baroreflex bradycardia in a dose-dependent manner [22]. The reduction in blood pressure can be explained by the suppression of sympathetic nerve activity, vasodilatation, and depression of myocardial contractility. In addition to its cardiovascular effects, propofol is a strong respiratory depressant with dose-dependent effects [23]. The mechanisms that explain the respiratory depressant effect of propofol have not been fully clarified yet. Kashigawi et al. found that propofol reduced the activity of inspiratory neurons located in the medulla by activation of the GABA_A_ receptors [24].

Similar to the findings in the literature, a significant decrease was observed in SBP, DBP, HR, and RR values in the group in which only propofol was administered in our study (Figure 2A, Figure 3A, Figure 4A and Figure 5A). All rats in this group died at the end of the 60-min period (Figure 1).

Despite the several hypotheses proposed to explain the mechanism of action of ILE therapy, none of them was able to bring a complete clarification. The first hypothesis on the mechanism of action suggests that ILE causes detachment of lipophilic drugs from the target tissues by forming lipid-rich compartments in the plasma (lipid sink) [25,26]. This scavenging effect is not only a static sink effect, but it is also considered as a dynamic shuttle effect. The lipid compartment in the blood creates the shuttle effect by removing the lipophilic drugs from the organs. In this way, organs with high blood flow are detoxified from the drug [27]. In one study, it has been shown that ILE therapy acutely decreased drug concentrations in the heart and brain but increased the concentrations in the liver [25].

Other hypotheses, suggested in order to explain the mechanism of action, are based on the cardiotonic and inotropic effects of ILE. One study reported that ILE administration increased the left ventricular contractility [28]. A study performed by Umpierrez et al. showed that ILE administration caused increases in blood pressure in African-American patients with type 2 diabetes [29]. Both the inotropic and lusitropic effects of ILE have been demonstrated in the absence of drug toxicity in cardiac studies in vivo and in isolated heart studies [30,31].

Huang et al. demonstrated that, similar to the agents opening the calcium (Ca) channels, long-chain fatty acids activated the voltage-gated Ca channels in isolated cardiomyocytes [32]. Another study observed that ILE showed an improvement of myocyte functions by increasing intracellular Ca levels [33]. However, there are other studies in the literature with contradicting findings. Xiao et al. conducted a study on Neonatal Rat Ventricular Myocytes to examine the role of polyunsaturated fatty acids (PUFAs) on l-type Ca channel currents and on the Ca release from the sarcoplasmic reticulum. They found that PUFAs reduced the intracellular Ca levels by both blocking the l-type Ca channel currents and decreasing the release of calcium from the sarcoplasmic reticulum [34]. In another study, ILE and verapamil were administered both in combination and alone individually, investigating the effects of l-type Ca channel activities and the intracellular Ca levels in the ventricular cardiomyocytes. The study found that ILE had no effects on the intracellular Ca levels and on the myocardial contractility in the absence of verapamil administration [35]. The validity of the calcium hypothesis has not been completely established yet and needs further study.

In our study, SBP, DBP, and HR values in the ILE+PP group started to decrease in the 5th min, the time-point when propofol was administered. However, after the 8th min, an increase was observed in SBP, DBP, and HR values; and these values got closer to those of the Control group. A similar course was detected in SBP, DBP, and HR values in the Control group after the 30th min. According to findings of this present study, decreased values of SBP, DBP, and HR associated with the administration of propofol, recovered with ILE administration. At this point, it is considered that ILE treatment is advantageous for preventing the untoward hypotension and bradycardia effects of propofol (Figure 2A, Figure 3A and Figure 5A).

Again, parallel to other studies, it was observed that RR values in the ILE+PP group had a decreasing course starting from the 5th min when propofol was started. After the 8th min, an increase was observed in RR values, getting closer to those in the Control group. A similar course was detected in RR values in the Control group after the 30th min. We observed that ILE treatment is also advantageous in preventing the respiratory depressant effects of propofol (Figure 4A).

The mortality rate was 100% in the PP group (Figure 1), whereas, the survival rate was found to be 100% in the ILE+PP group. Our study is important as it shows that ILE therapy decreased propofol-related mortality. However, it is not possible to compare our results with those of previous studies since ILE therapy was not investigated before whether it would prevent propofol-induced mortality.

According to our findings, the RR level of the ILE group was found to be significantly higher than those of the other groups (Table 1, Figure 4A). Further studies should be conducted to explain the tachypneic effect of ILE therapy in detail.

## 5. Conclusions

Our study findings led us to the conclusion that side effects—such as hypotension, bradycardia, and respiratory depression; which can be seen after propofol application in rats—could be recovered with ILE therapy; and mortality due to these side effects would be prevented. Further studies with more extensive designs are required.

## Figures and Tables

**Table 1:** Differences of least squares means (MD) and adjusted *p* (Adj *p*) values of all pairwise comparisons among groups (1: Control, 2: PP, 3:ILE, 4: ILE+PP).

	1–2		1–3		1–4		2–3		2–4		3–4	
Scores	MD	Adj P	MD	Adj P	MD	Adj P	MD	Adj P	MD	Adj P	MD	Adj P
SBP (mmHg)	**36.78**	*<0.0001*	0.38	*1.000*	**7.80**	*0.009*	**−36.40**	*<0.0001*	**−28.98**	*<0.0001*	**7.42**	*0.013*
DBP (mmHg)	**24.63**	*<0.0001*	1.17	*1.000*	−3.78	*1.000*	**−23.46**	*<0.0001*	**−28.41**	*<0.0001*	−4.95	*0.937*
RR (/min)	**14.11**	*<0.0001*	**−13.13**	*<0.0001*	1.07	*1.000*	**−27.25**	*<0.0001*	**−13.05**	*<0.0001*	**14.20**	*<0.0001*
HR (beats/min)	**135.14**	*<0.0001*	−24.08	*0.183*	22.85	*0.234*	**−159.23**	*<0.0001*	**−112.29**	*<0.0001*	**46.93**	*0.0012*

Bonferroni’s correction was used for multiple comparisons. SBP: systolic blood pressure, DBP: diastolic blood pressure, RR: respiratory rate, HR: heart rate.

## References

[1] Shafer A., Doze V.A., Shafer S.L., White P.F. (1988). Pharmacokinetics and pharmacodynamics of propofol infusions during general anesthesia. Anaesthesiolsthesiology.

[2] Hug J.C., McLeskey C.H., Nahrwold M.L., Roizen M.F., Stanley T.H., Thisted R.A., Grasela T.H. (1993). Hemodynamic effects of propofol: Data from over 25,000 patients. Anesth. Analg..

[3] Zahoor A.B., Ahmed N.A. (2010). The effects of duration of Propofol injection on hemodynamics. Middle East J. Anaesthesiol..

[4] Ebert T.J. (2005). Sympathetic and hemodynamic effects of moderate and deep sedation with propofol in humans. Anaesthesiol. J. Am. Soc. Anaesthesiol..

[5] Weinberg G., Ripper R., Feinstein D.L., Hoffman W. (2003). Lipid emulsion in fusion rescues dogs from bupivacaine-induced cardiactoxicity. Reg. Anesth. Pain Med..

[6] Weinberg G.L., VadeBoncouer T., Ramaraju G.A., Garcia-Amaro M.F., Cwik M.J. (1998). Pretreatment or Resuscitation with a Lipid Infusion Shifts the Dose-Response to Bupivacaine-induced Asystole in Rats. Anaesthesiolsthesiology.

[7] Cevik S.E., Tasyurek T., Guneysel O. (2014). Intralipid emulsion treatment as an antidote in lipophilic drug intoxications. Am. J. Emerg. Med..

[8] Cave G., Harvey M.G., Castle C.D. (2006). The role of fat emulsion therapy in a rodent model of propranolol toxicity: A preliminary study. J. Med. Toxicol..

[9] Kang C., Kim D.H., Kim S.C., Lee S.H., Jeong J.H., Kang T.S., Shin I.W., Kim R.B., Lee D.H. (2015). The effects of intravenous lipid emulsion on prolongation of survival in a rat model of calcium channel blocker toxicity. Clin. Toxicol..

[10] Tsujikawa S., Matsuura T., Hori K., Mori T., Kuno M., Nishikawa K. (2018). Superior efficacy of lipid emulsion infusion over serum alkalinization in reversing amitriptyline-induced cardiotoxicity in Guinea Pig. Anesth. Analg..

[11] Rothschild L., Bern S., Oswald S., Weinberg G. (2010). Intravenous lipid emulsion in clinical toxicology. Scand. J. Trauma Resusc. Emerg. Med..

[12] Tsui B.C., Cheung S.S., Ziwenga O., Gragasin F.S. (2015). Use of Intralipid^®^ in managing refractory hypotension following epidural blockade. Can. J. Anesth..

[13] Harkness J.E., Turner P.V., VandeWoude S., Wheler C.L. (2013). Harkness and Wagner’s Biology and Medicine of Rabbits and Rodents.

[14] Turan C.A., Ozturk T.C., Akoglu E.U., Ak R., Aygun K., Sahiner A., Onur O.E. (2018). The role of intralipid emulsion in the rat model of digoxin intoxication. Cardiovasc. Toxicol..

[15] Cleophas T.J., Zwinderman A.H. (2012). Statistics Applied to Clinical Studies.

[16] O’Connell N.S., Dai L., Jiang Y., Speiser J.L., Ward R. (2017). Methods for Analysis of Pre-Post Data in Clinical Research: A Comparison of Five Common Methods. J. Biom. Biostat..

[17] Stoetzer C., Reuter S., Doll T., Foadi N., Wegner F., Leffler A. (2016). Inhibition of the cardiac Na+ channel α-subunit Nav1. 5 by propofol and dexmedetomidine. Naunyn-Schmiedeberg’s Arch. Pharmacol..

[18] Friederich P., Benzenberg D., Urban B.W. (2002). Ketamine and Propofol Differently Inhibit Human Neuronal K^+^ Channels. Eur. J. Anaesth..

[19] Cook D.J., Housmans P.R. (1994). Mechanism of the negative inotropic effect of propofol in isolated ferret ventricular myocardium. Anaesthesiolsthesiology.

[20] Riou B., Besse S., Lecarpentier Y., Viars P. (1992). In vitro effects of propofol on rat myocardium. Anaesthesiolsthesiology.

[21] Claeys M., Gepts E., Camu F. (1988). Haemodynamic changes during anaesthesia induced and maintained with propofol. Br. J. Anaesth..

[22] Ebert J.T., Muzi M., Berens R., Goff D., Kampine P.J. (1992). Sympathetic responses to induction of anesthesia in humans with propofol or etomidate. Anaesthesiolsthesiology.

[23] Goodman N.W., Black A.M.S., Carter J.A. (1987). Some ventilatory effects of propofol as sole anaesthetic agent. Br. J. Anaesth..

[24] Kashiwagi M., Osaka Y., Onimaru H., Takeda J. (2011). Optical imaging of propofol-induced central respiratory depression in medulla–spinal cord preparations from new born rats. Clin. Exp. Pharmacol. Physiol..

[25] Shi K., Xia Y., Wang Q., Wu Y., Dong X., Chen C., Papadimos T.J. (2013). The effect of lipid emulsion on pharmacokinetics and tissue distribution of bupivacaine in rats. Anesth. Analg..

[26] Litonius E., Tarkkila P., Neuvonen P.J., Rosenberg P.H. (2012). Effect of intravenous lipid emulsion on bupivacaine plasma concentration in humans. Anaesthesia.

[27] Fettiplace M.R., Weinberg G. (2018). The mechanisms underlying lipid resuscitation therapy. Reg. Anesth. Pain Med..

[28] Holland D.J., Erne D., Kostner K., Leano R., Haluska B.A., Marwick T.H., Sharman J.E. (2011). Acute elevation of triglycerides increases left ventricular contractility and alters ventricular-vascular interaction. Am. J. Physiol. Heart Circ. Physiol..

[29] Umpierrez G.E., Smiley D., Robalino G., Peng L., Kitabchi A.E., Khan B., Phillips L.S. (2009). Intravenous intralipid-induced blood pressure elevation and endothelial dysfunction in obese African-Americans with type 2 diabetes. J. Clin. Endocrinol. Metab..

[30] Shin I.W., Hah Y.S., Kim C., Park J., Shin H., Park K.E., Lim D.H. (2014). Systemic blockage of nitric oxide synthase by L-NAME increases left ventricular systolic pressure, which is not augmented further by Intralipid^®^. Int. J. Biol. Sci..

[31] Fettiplace M.R., Ripper R., Lis K., Lin B., Lang J., Zider B., Weinberg G. (2013). Rapid cardiotonic effects of lipid emulsion infusion. Crit. Care Med..

[32] Huang J.M., Xian H., Bacaner M. (1992). Long-chain fatty acids activate calcium channels in ventricular myocytes. Proc. Natl. Acad. Sci. USA.

[33] Park J., Kim Y.A., Han J.Y., Jin S., Ok S.H., Sohn J.T., Shin I.W. (2016). Lipofundin^®^ MCT/LCT 20% increase left ventricular systolic pressure in an ex vivo rat heart model via increase of intracellular calcium level. Korean J. Anaesthesiol..

[34] Xiao Y.F., Gomez A.M., Morgan J.P., Lederer W.J., Leaf A. (1997). Suppression of voltage-gated L-type Ca^2+^ currents by polyunsaturated fatty acids in adult and neonatal rat ventricular myocytes. Proc. Natl. Acad. Sci. USA.

[35] Kryshtal D.O., Dawling S., Seger D., Knollmann B.C. (2016). In vitro studies indicate intravenous lipid emulsion acts as lipid sink in verapamil poisoning. J. Med. Toxicol..

